# Essentiality Assessment of Cysteinyl and Lysyl-tRNA Synthetases of *Mycobacterium smegmatis*

**DOI:** 10.1371/journal.pone.0147188

**Published:** 2016-01-21

**Authors:** Sudha Ravishankar, Anisha Ambady, Rayapadi G. Swetha, Anand Anbarasu, Sudha Ramaiah, Vasan K. Sambandamurthy

**Affiliations:** 1 AstraZeneca India Pvt Ltd, Bellary Road, Hebbal, Bengaluru, 560024, India; 2 School of Biosciences & Technology, VIT University, Vellore, 632014, India; Institute of Molecular Genetics IMG-CNR, ITALY

## Abstract

Discovery of mupirocin, an antibiotic that targets isoleucyl-tRNA synthetase, established aminoacyl-tRNA synthetase as an attractive target for the discovery of novel antibacterial agents. Despite a high degree of similarity between the bacterial and human aminoacyl-tRNA synthetases, the selectivity observed with mupirocin triggered the possibility of targeting other aminoacyl-tRNA synthetases as potential drug targets. These enzymes catalyse the condensation of a specific amino acid to its cognate tRNA in an energy-dependent reaction. Therefore, each organism is expected to encode at least twenty aminoacyl-tRNA synthetases, one for each amino acid. However, a bioinformatics search for genes encoding aminoacyl-tRNA synthetases from *Mycobacterium smegmatis* returned multiple genes for glutamyl (GluRS), cysteinyl (CysRS), prolyl (ProRS) and lysyl (LysRS) tRNA synthetases. The pathogenic mycobacteria, namely, *Mycobacterium tuberculosis* and *Mycobacterium leprae*, were also found to possess two genes each for CysRS and LysRS. A similar search indicated the presence of additional genes for LysRS in gram negative bacteria as well. Herein, we describe sequence and structural analysis of the additional aminoacyl-tRNA synthetase genes found in *M*. *smegmatis*. Characterization of conditional expression strains of Cysteinyl and Lysyl-tRNA synthetases generated in *M*. *smegmatis* revealed that the canonical aminoacyl-tRNA synthetase are essential, while the additional ones are not essential for the growth of *M*. *smegmatis*.

## Introduction

In bacterial cells, like transcription and replication, translation is one of the key processes which leads to the synthesis of every protein required for the cell to function irrespective of their final localization [1]. Translation is a multi-step process involving several components like tRNAs, amino acids, mRNA, ribosomes. A large number of antibiotics discovered till date are inhibitors of translation, thereby establishing the importance of this process for bacterial cell survival [2]. Each of these antibiotic seems to affect a unique step in the protein synthesis cascade [3]. The bacterial growth inhibition observed with mupirocin is brought about by the inhibition of isoleucyl-tRNA synthetase (IleRS) which in turn leads to the inhibition of translation process [4]. The clinical success of mupirocin as a topical antibiotic [4] has opened up attractive opportunities to target aminoacyl-tRNA synthetases (aaRS) for novel antibacterial agents. Several attempts that were made, including a few *in silico* search and design [5, 6] have led to the discovery of inhibitors for a number of aaRS such as PheRS [7, 8], MetRS [9, 10], ProRS [11], TyrRS [12], LeuRS [5, 13], ThrRS [14]. Ochsner *et al*., have presented a comprehensive review of all the known aaRS inhibitors [15]. Additionally, 4-thiozolidinone derivatives, Microcin C and tobramycin are known to inhibit AspRS [16]. Although, there is a good degree of similarity between eukaryotic and prokaryotic aaRS, identification of bacteria selective inhibitors has provided ample evidence to discover novel, selective inhibitors of this enzyme class.

During translation, each amino acid is carried by a specific tRNA to the translation site. These tRNAs get charged with respective amino acids by the action of aminoacyl-tRNA synthetases or aminoacyl-tRNA ligases in a two-step process [17]. Therefore, synthesis of aminoacyl-tRNA (aa-tRNA) is a critical step in translation and hence aaRS are considered essential for bacterial survival. The mechanism of this class of enzymes suggest that each cell should possess at least twenty aaRS as there are 20 different natural amino acids [17]. However, it has been observed that this number is either more than 20 or less than 20 in a few organisms [18]. The aaRS are generally divided into two classes, I and II, based on their structural features. In all of the aaRS studied, the tRNA binding region has a conserved α-helical structure. The class I enzymes are generally monomeric, share a characteristic Rossman-fold catalytic domain and two conserved motifs, HIGH and KMSKS. On the other hand, the class II aaRS are dimeric or multimeric, contain at least three conserved regions and share an anti-parallel β-sheet fold flanked on either side by α-helices. Another catalytic difference between these two classes of enzymes is that, the class I enzymes couple the aminoacyl group to the 2'-hydroxyl of the last nucleotide of tRNA, while, the class II enzymes couple aminoacyl group to the 3'-hydroxyl of the last nucleotide of tRNA [19, 20].

In general, bacteria are believed to possess at least one aaRS for each amino acid in order to supply the translation machinery with the respective aa-tRNA. Interestingly, some organisms seem to carry more than one gene coding for the same aaRS. For example, *Escherichia coli* encodes two lysyl-tRNA synthetase genes, one is expressed constitutively, while the other is inducible [21]. *E*. *coli* also codes for two glutamyl-tRNA synthetases, where one uses cognate tRNA^Glu^ [22], while the other one uses tRNA^Asp^ as substrate to transfer the activated glutamyl group [23]. A query in KEGG genes database for genes coding for ‘tRNA synthetases’ of *M*. *smegmatis* returned a list of 24 genes with multiple genes coding for glutamyl (MSMEG_2383, MSMEG_6306), prolyl (MSMEG_2621, MSMEG_5671), cysteinyl (MSMEG_4189, MSMEG_6074) and lysyl (MSMEG_3796, MSMEG_6094) tRNA synthetases [24]. A similar search against *M*. *tuberculosis and M*. *leprae* identified 2 genes each for lysyl and cysteinyl-tRNA synthetases. However, these organisms were found to have only one gene for glutamyl and prolyl-tRNA synthetase. Multiple sequence alignment of the *M*. *smegmatis* cysteinyl and lysyl-tRNA synthetases with those of *M*. *tuberculosis* and *M*. *leprae* revealed that the respective orthologs have a high degree of sequence similarity. The essentiality of lysyl and cysteinyl-tRNA synthetases of *M*. *smegmatis* were evaluated by employing conditional expression strains generated in *M*. *smegmatis* using the isopropylthiogalactoside (IPTG) inducible conditional expression system [25].

## Materials and Methods

### Bacterial strains, media, chemicals and reagents

Bacterial strains used in this study are listed in Table 1. Glycerol, Tween 80, kanamycin and IPTG were purchased from SIGMA, USA. Restriction enzymes, Taq DNA polymerase, DNA ladders were purchased from New England Biolabs, USA. Hygromycin B was obtained from Roche, Fusion polymerase from Finnzymes. Pristinamycin was obtained from Sanofi Aventis and the P_1_ component of pristinamycin was purified in-house as described earlier [26]. Luria Bertani (LB) broth and LB agar were used to grow *E*. *coli*. Middlebrook 7H9 (DIFCO) supplemented with 0.2% Glycerol (v/v), 0.05% Tween 80 (w/v) and albumin-dextrose was used for growing broth cultures of mycobacteria and Middlebrook 7H11 (DIFCO) for measurement of colony forming units (CFU). Bacterial cultures were supplemented with antibiotics, IPTG and P_1_ as required.

**Table 1:** List of plasmids and strains used in this study.

Plasmids/ Strains	Reference	Details
pAZI9452^$^	25*	Conditional expression vector with an IPTG-inducible promoter
pAZI9479^$^	26*, 27*	Conditional expression vector with a pristinamycin-inducible promoter system
pMV261	28*	An *E*. *coli*–mycobacterial shuttle vector for protein expression in mycobacteria driven by hsp60 promoter.
pAZI9501	This study	Conditional expression plasmid of *M*. *smegmatis leuS* in pAZI9452
pAZI9502	This study	Conditional expression plasmid of *M*. *smegmatis leuS* in pAZI9479
pAZI9503	This study	Conditional expression plasmid of *M*. *tuberculosis leuS* in pAZI9479
pAZI9504	This study	Conditional expression plasmid of MSMEG_6074 in pAZI9452
pAZI9505	This study	Conditional expression plasmid of MSMEG_6073 in pMV261
pAZI9506	This study	Conditional expression plasmid of MSMEG_3796 in pAZI9452
pAZI9507	This study	Conditional expression plasmid of MSMEG_6094 in pAZI9452
SleuS/KD-I	This study	Conditional expression strain of *M*. *smegmatis leuS* with an IPTG-inducible system
SleuS/KD-P	This study	Conditional expression strain of *M*. *smegmatis leuS* with pristinamycin-inducible system
TleuS/KD-P	This study	Conditional expression strain of *M*. *tuberculosis leuS* with a pristinamycin-inducible system
ScysS/KD-I	This study	Conditional expression strain of *M*. *smegmatis cysS* (MSMEG_6074) with an IPTG-inducible system
ScysS/KD-I-compl	This study	*M*. *smegmatis* cysS conditional expression strain complemented with pBAN6073
S3796/KD-I	This study	Conditional expression strain of *M*. *smegmatis* MSMEG_3796 with an IPTG-inducible system
S6094/KD-I	This study	Conditional expression strain of *M*. *smegmatis* MSMEG_6094 with an IPTG-inducible system
*E*. *coli* DH5α	Lab. stock	*endA1*, *hsdR17*, *supE44*, *recA1*, *relA1*, (*lacZYA- argF*)
*M*. *smegmatis* mc^2^155	Lab. stock	Non-pathogenic mycobacterial strain.
*M*. *tuberculosis* H37Rv 27294	Lab. stock	Virulent strain of *M*. *tuberculosis* H37Rv

*References

^$^ Fig A in S1 File

### Generation of conditional expression plasmids

All plasmids used in this study are listed in Table 1. Conditional expression plasmids were generated by cloning about 700 bps of DNA fragment of each target gene amplified from its 5’ end. The primers listed in Table A in S1 File were used for generating required amplicons. Fusion polymerase was used to generate amplicons in a 25-cycle polymerase chain reaction with cycling conditions of denaturing at 98°C, annealing at a temperature dictated by the melting temperature of each primer pair (Table A in S1 File) and extension at 72°C. All the recombinant plasmids constructed on pAZI9452 [25] background (pAZI9501, pAZI9504, pAZI9506 and pAZI9507) were generated by cloning the respective amplicons at NdeI and HindIII sites of pAZI9452. Two of the recombinant plasmids, pAZI9502 and pAZI9503 were generated by cloning the respective amplicons at NcoI and MscI sites of pAZI9479 [26, 27].

In *M*. *smegmatis*, *cysS* is present in an operon where MSMEG_6073 is the last gene after *cysS*. MSMEG_6073 was PCR amplified using primers S6073complF and S6073complR (Table A in S1 File) and cloned into pMV261 [28] vector at BamHI and HindIII sites to generate pAZI9505. The recombinant plasmids were screened by restriction enzyme analysis and sequence of the cloned fragments were confirmed by DNA sequencing.

### Generation of conditional expression strains

Conditional expression plasmids were electroporated into *M*. *smegmatis* mc^2^155 or *M*. *tuberculosis* H37Rv following a standard protocol [29]. The transformation mix was plated onto 7H9 agar plates containing 50 μg/ml hygromycin and supplemented with either 500 μM IPTG or 300 ng/ml of P_1_. The colonies were screened for their dependence on inducer for growth by replica plating on 7H11 plates with and without the inducer. The genotype of these recombinants (presence of truncated version of the gene of interest downstream of the native promoter and the full length gene downstream of an inducible promoter—depicted in Fig B in S1 File) were confirmed by PCR as described earlier [25, 26] using the primers listed in Table A in S1 File. The colonies that were positive by both screens were designated as SleuS/KD-I, ScysS/KD-I, S3796/KD-I and S6094/KD-I for the conditional expression strains of *M*. *smegmatis leuS*, *cysS*, *MSMEG_3796*, *MSMEG_6094*, respectively, on a pAZI9452 vector backbone. Similarly, conditional expression strains generated with pAZI9479 of *M*. *tuberculosis leuS* and *M*. *smegmatis leuS* were designated as TleuS/KD-P and SleuS/KD-P, respectively. Subsequently, ScysS/KD-I was electroporated with pAZI9505 to generate a complemented strain ScysS/KD-I/C.

### Analysis of inducer dependency of conditional expression strains

Each of the PCR confirmed conditional expression strain was grown in 2 ml of 7H9 broth supplemented with either 500 μM IPTG or 300 ng/ml P_1_ as appropriate. When the cultures reached mid-logarithmic phase, they were centrifuged and the harvested cells were washed with 7H9 broth followed by resuspension in fresh broth to be used as inoculum. In order to determine the minimum inducer concentration required for growth of each of the conditional expression strain, several dilutions of the culture inoculum were either plated or spotted on 7H11 plates containing different concentration of inducer i.e., 0–500 μM IPTG or 0–300 ng/ml of P_1_. *M*. *smegmatis* mc^2^155 and *M*. *tuberculosis* H37Rv were also processed in a similar way and plated on 7H11 plates to compare the colony morphology and growth rate. Minimum inducer required for the growth of each conditional expression strain was identified as the concentration at which the conditional expression strain grew as well as the wild-type strain. Subsequently, the conditional expression strains were grown at the identified inducer concentration until they reached mid-log phase, the cells were washed and used to prepare inoculum for further experiments.

### Protein sequence resource

KEGG genes database at http://www.genome.jp/kegg/genes.html was used to retrieve the information regarding all the tRNA synthetases present in *E*. *coli*, *Haemophilus influenzae*, *Staphylococcus aureus*, *Streptococcus pneumoniae*, *M*. *smegmatis*, *M*. *tuberculosis* and *M*. *leprae*. The same database was used to retrieve DNA sequences of the required aminoacyl-tRNA synthetases for cloning purposes and protein sequences for sequence homology analysis.

### Transcription unit

*M*. *smegmatis* mc^2^155 database from BioCyc genome pathway database collection (http://biocyc.org/MSME246196/organism-summary?object=MSME246196) was referred for analysing the transcription unit arrangement of each gene.

### Pairwise sequence alignment

The required protein sequences were retrieved from KEGG genes database. SIM protein sequence alignment tool in the ExPASy Bioinformatics Resource Portal (http://web.expasy.org/sim/) was used to analyse the percent identity between the selected protein sequences.

### Protein database search

NCBI BLASTP program (http://blast.ncbi.nlm.nih.gov/Blast.cgi?PAGE=Proteins) was used to search the non-redundant protein sequence database for homologous protein sequences and/or the probable protein families the query sequence belongs to.

### Transmembrane segment prediction analysis

TMHMM v 2.0 (http://www.cbs.dtu.dk/services/TMHMM/) at the Centre for Biological Sequence Analysis (CBS) was used to predict the transmembrane segments present in MSMEG_3796, MSMEG_6094 and *S*. *aureus* MprF.

### Protein structure analysis

#### 3-Dimensional model structure generation

The protein sequence of MSMEG_5671 was submitted to an online server, I-TASSER (http://zhanglab.ccmb.med.umich.edu/I-TASSER/) which generated 5 model structures. A model with highest confidence score was selected for further structural analysis. The 3-dimensional (3-D) structure of *E*. *coli* YbaK was downloaded from PDB (PDB-ID: 2DXA).

#### Assessment of modelled MSMEG_5671 structure

The structure verification of MSMEG_5671 was performed using the UCLA Structure Analysis and Verification Server with PROCHECK, ERRAT and VERIFY-3D programs (http://services.mbi.ucla.edu/SAVES/).

#### Pairwise structure comparison

A pairwise structure comparison was performed between the modelled structure of *M*. *smegmatis* MSMEG_5671 and *E*. *coli* YbaK structure using DaliLite server (http://www.ebi.ac.uk/Tools/structure/dalilite/) which uses sum-of-pairs method by comparing the intramolecular distance matrices and produces a measure of similarity.

## Results and Discussion

### Multiple aaRS of *M*. *smegmatis*

Aminoacyl-tRNA synthetases are one of the key players in the translation process [17]. They catalyse the coupling of an aminoacyl group to its cognate tRNA in a two-step process (Fig C in S1 File), in the first step an amino acid gets activated which is transferred to its cognate tRNA in the second step. Thus, all the cognate aaRS would possess two major domains, a catalytic domain where the aminoacyl-tRNA is synthesized and a tRNA anticodon recognition domain. Some of the aaRS also have a built-in editing domain needed to remove the amino acids from an erroneously charged tRNA [30].

A query in KEGG genes database for a list of genes encoding ‘tRNA synthetase’ of *M*. *smegmatis* returned 24 genes [24]. Like other mycobacteria, *M*. *smegmatis* also does not code for AsnRS and GlnRS and hence should have 18 genes coding for the other 18 essential aaRS [16]. Among the 24 genes found to code for the various aaRS, PheRS is encoded by 2 genes, one coding for α and the other for the β subunit of this enzyme. Four out of the other five additional genes were found to code for an additional glutamyl, cysteinyl, prolyl and lysyl-tRNA synthetases, respectively, while the fifth additional gene *tilS* is a tRNA^Ile^ lysidine synthetase (Table 2). A similar search in KEGG for the pathogenic mycobacteria, *M*. *tuberculosis* and *M*. *leprae* returned a list with 20 and 21 aaRS entries, respectively. The list indicated the presence of an additional CysRS and LysRS but not GluRS and ProRS (Table 2). Results from search performed against two Gram positive bacteria (*S*. *aureus* and *S*. *pneumoniae*) and two Gram negative bacteria (*E*. *coli* and *H*. *influenzae*) showed the presence of additional genes for lysyl-tRNA synthetase in Gram negative bacteria only (Table 2).

**Table 2:** Genes encoding aminoacyl-tRNA synthetases*.

	gene/s encoding aminoacyl-tRNA synthetases in						
aaRS	*M*. *smegmatis*	*M*. *tuberculosis*	*M*. *leprae*	*E*. *coli*	*H*. *influenzae*	*S*. *aureus*	*S*. *pneumoniae*
AlaRS	MSMEG_3025	Rv2555c	ML0512	b2697	HI0814	SA1446	SP1383
GlyRS	MSMEG_4485	Rv2357c	ML0826	b3559,b3560	HI0924,HI0927	SA1394	SP1474,SP1475
ValRS	MSMEG_4630	Rv2448c	ML1472	b4258	HI1391	SA1488	SP0568
LeuRS	MSMEG_6917	Rv0041	ML0032	b0642	HI0921	SA1579	SP0254
IleRS	MSMEG_3169	Rv1536	ML1195	b0026	HI1586	SA1036	SP1659
TilS	MSMEG_6111	-	-	b0188	-	-	-
SerRS	MSMEG_6413	Rv3834c	ML0082	b0893	NI0110	SA0009	SP0411
ThrRS	MSMEG_2931	Rv2614c	ML0456	b1719	HI1367	SA1506	SP1631
ProRS	MSMEG_2621MSMEG_5671	Rv2845c	ML1553	b0194	HI0729	SA1106	SP0264
TrpRS	MSMEG_1657	Rv3336c	ML0686	b3384	HI0637	SA0855	SP2229
TyrRS	MSMEG_3758	Rv1689	ML1352	b1637	HI1610	SA1550	SP2100
PheRS	MSMEG_3777,MSMEG_3778	Rv1649,Rv1650	ML1401,ML1402	b1713,b1714	HI1311,HI1312	SA0985,SA0986	SP0579,SP0581
MetRS	MSMEG_5441	Rv1007c	ML0238	b2114	HI1276	SA0448	SP0788
CysRS	MSMEG_4189,MSMEG_6074	Rv3580c,Rv2130c	ML0323,ML1302	b0526	HI0078	SA0488	SP0591
AspRS	MSMEG_3003	Rv2572c	ML0501	b1866	HI0317	SA1456	SP2114
GluRS	MSMEG_2383,MSMEG_6306	Rv2992c	ML1688	b0144,b2400	HI0274	SA0486	SP2069
AsnRS	-	-	-	b0930	HI1302	SA1287	SP1542
GlnRS	-	-	-	b0680	HI1354	-	-
LysRS	MSMEG_3796,MSMEG_6094	RV3598c,Rv1640c	ML1393,ML0233	b2890,b4129	HI0836,HI1121	SA0475	SP0713
HisRS	MSMEG_2976	Rv2580c	ML0494	b2514	HI0369	SA1457	SP2121
ArgRS	MSMEG_4959	Rv1292	ML1127	b1876	HI1583	SA0564	SP2078

*KEGG genes database

### Sequence analysis of tRNA^Ile^ lysidine synthetase and glutamyl-tRNA synthetase

In the absence of genetic and biochemical characterization for these aaRSs from *M*. *smegmatis*, pairwise sequence alignment was performed to understand the degree of homology with their orthologs from *M*. *tuberculosis* and *E*. *coli*.

MSMEG_6111, annotated to code for tRNA^Ile^ Lysidine synthetase was found to be 34% identical to the *E*. *coli* TilS. A search for homologs of MSMEG_6111 identified the presence of orthologs (annotated as *mesJ*) in all the other organisms analysed in this study. Although, experimental evidence is required to establish the physiological role and essentiality of MSMEG_6111 in *M*. *smegmatis*, it’s orthologs from *E*. *coli* and *M*. *tuberculosis* were demonstrated to be essential [31, 32]. Biochemical characterization of the *E*. *coli* ortholog has led to the understanding that Tils is needed to synthesize tRNA^Ile^ Lysidine in a reaction where the CAU anticodon of tRNA^Ile^ gets converted to LAU when the enzyme transfers a lysine moiety on to the cytidine residue present on the anticodon. This activity is required to maintain translation fidelity because it prevents mis-charging of tRNA^Ile^ by MetRS [33, 34].

*M*. *smegmatis* was found to possess two *gltX* genes, one coding for glutamyl-tRNA synthetase (GluRS) and another one for glutamyl-Q-tRNA^Asp^ synthetase (Glu-Q-RS), similar to those in *E*. *coli*. Pairwise sequence alignment of *M*. *smegmatis* GluRS and Glu-Q-RS indicated only about 39% identity between them in their N-terminal 250 amino acids, a result similar to the one observed between *E*. *coli* GluRS and Glu-Q-RS. However, Only GluRS orthologs could be found in other mycobacterial species and the gram positive and gram negative bacteria analysed in this study. In *E*. *coli*, both GluRS and Glu-Q-RS were shown to activate glutamate. While the activated glutamate is transferred to tRNA^Glu^ by GluRS to synthesize Glu-tRNA^Glu^, Glu-Q-RS transfers it to tRNA^Asp^ to synthesize Glu-tRNA^Asp^ [35, 36]. Glu-Q-RS was considered non-essential as its product Glu-tRNA^Asp^ could not bind EF-Tu to participate in the translation process. This was substantiated later by the experimentally derived essential and dispensable nature of GluRS and Glu-Q-RS respectively in *E*. *coli* [37, 38]. Although, the situation could be very similar in *M*. *smegmatis*, biochemical and genetic evidence is required to establish the role and essentiality of *M*. *smegmatis* GluRS and Glu-Q-RS. However, the evolutionary significance of the conservation of the non-essential Glu-Q-RS across different bacterial genera has remained unclear.

### Sequence and structural analysis of prolyl-tRNA synthetase

Both MSMEG_2621 and MSMEG_5671 have been annotated as Prolyl-tRNA synthetase in the KEGG genes database. MSMEG_2621, coding for a 585 amino acid prolyl-tRNA synthetase has been indicated to possess motifs for a catalytic, an anti-codon binding and a tRNA editing activity, similar to the ones found in the canonical prolyl-tRNA synthetase of *E*. *coli* and *M*. *tuberculosis*. On the other hand, MSMEG_5671, which codes for a 159 amino acid protein has been suggested to contain a tRNA editing motif. BlastP analysis of MSMEG_5671 protein sequence indicated that it belongs to YbaK-like superfamily of proteins with homologs present in several bacterial genera. YbaK, an *E*. *coli* protein annotated as Cys-tRNA (Pro)/Cys-tRNA (Cys) deacylase has been shown to deacylate the mischarged Cys-tRNA^Pro^ to keep tRNA^Pro^ available for acylation with proline [39]. In a similar reaction, INS domain present within the canonical ProRS has been shown to deacylate mischarged Ala-tRNA^Pro^ to make the tRNA^Pro^ available for acylation with proline [40, 41]. Thus, the two editing motifs, one cis-acting INS domain and the other trans-acting YbaK like protein deacylate the wrongly charged tRNA^Pro^, help the cells to maintain translation fidelity [42]. Surprisingly, no significant homology to any part of MSMEG_2621 was observed when MSMEG_5671 was aligned with MSMEG_2621 in spite of both having tRNA editing motifs. Even the sequence identity between MSMEG_5671 and *E*. *coli* YbaK was only 27% over a stretch of 85 amino acids. Therefore, we decided to generate a 3-D model structure of MSMEG_5671 and compare it with the *E*. *coli* YbaK structure available in PDB.

### MSMEG_5671 structure prediction analysis

Crystal structures of *H*. *influenzae* YbaK at 1.8Å resolution and *E*. *coli* YbaK at 1.58Å resolution have been reported previously [43, 44]. The non-availability of an experimental 3-dimensional (3-D) structure of MSMEG_5671 prompted us to construct a 3-D model using its amino acid sequence. The I-TASSER server [45, 46] used for this purpose generated ten templates and the one with highest Z-score value of 3.5 (PDB ID: 2CX5, chain A) was taken as the best template based on which five model structures were produced with a reported TM score of 0.85±0.08. A model with a confidence score of 1.03 was selected as the best model for MSMEG_5671. The Ramachandran plot obtained using PROCHECK program [47] showed that 86.6% and 13.4% of residues in the modelled structure of MSMEG_5671 were in favored and allowed regions, respectively. The ERRAT [48] score of 94.702 was found to fall within the range of a high quality model suggesting that the backbone conformation and non-bonded interactions in the model were reasonable. VERIFY-3D [49] results showed that 91.82% of the residues in the modeled structure have an average score of >0.2 (Fig D in S1 File), thereby, confirming the model to be of good quality. Thus, evaluation of the MSMEG_5671 modelled structure through PROCHECK, ERRAT and VERIFY-3D programs established that the model possessed high geometric quality and a good energy profile.

### MSMEG_5671 and *E*. *coli* YbaK have comparable 3-D structure

**Fig 1:**
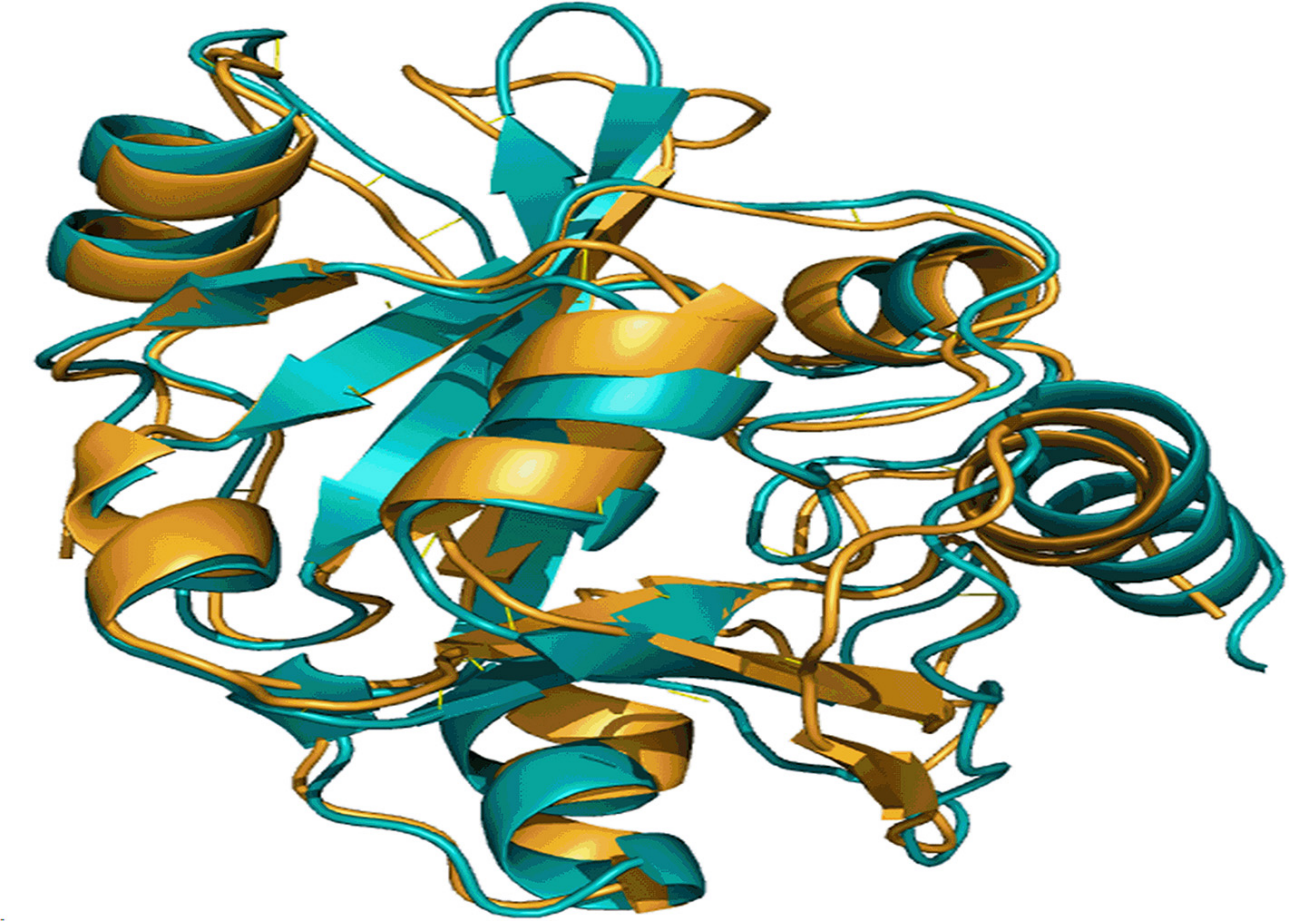
A snapshot of structural alignment of MSMEG_5671 and *E*. *coli* YbaK. Structure of *E*. *coli* YbaK (gold) and the modeled structure of MSMEG_5671 (cyan) were superimposed using DALI server.

In the absence of biochemical evidence, meaningful alignments generated through 3-D structure comparison methods enable understanding proteins and their functions. A pairwise structure comparison performed with MSMEG_5671 and *E*. *coli* YbaK using DALI server showed that the two structures align well with a Dali-Z score of 21.7 [50]. Superimposition of the two structures as depicted in Fig 1 demonstrated that they possess similar folds indicating that they could be performing same function. Thus, the generation of a 3-D model structure for MSMEG_5671 and comparing it with the structure of *E*. *coli* YbaK have clearly indicated that MSMEG_5671 is the most likely candidate performing Cys-tRNA^Pro^ deacylase activity in *M*. *smegmatis*.

The search in KEGG genes database for aminoacyl-tRNA synthetase genes did not yield additional prolyl-tRNA synthetase genes in *M*. *tuberculosis* and *M*. *leprae*. However, a careful examination of the list of orthologs of MSMEG_5671 in KEGG indicated presence of its homologs in these pathogenic organisms as well. Two proteins, RVBD_3224B (72 amino acids) of *M*. *tuberculosis* H37Rv and ML0799 (135 amino acids) of *M*. *leprae* have been currently annotated as hypothetical proteins and about 40% identical over a stretch of 50 residues and 30% identical over a stretch of 84 residues, respectively with MSMEG_5671. The generation of experimental data is required to prove that these proteins perform Cys-tRNA^Pro^ deacylation despite being shorter than YbaK and MSMEG_5671.

### Sequence analysis of cysteinyl-tRNA synthetase

Among the several organisms analysed in this study for cysteinyl-tRNA synthetases, the two Gram positive and two Gram negative bacteria were found to have a single gene each encoding this enzyme. However, all the three mycobacterial species had two genes each (Table 2). Unlike GluRS and ProRS, the two CysRS of *M*. *smegmatis* were of similar size (MSMEG_4189–412 and MSMEG_6074–477 amino acids). A pairwise sequence alignment of these two proteins revealed only about 37% identity between them in their N-terminal 300 amino acids stretch indicating existence of significant sequence differences in their C-terminal portion. Chemical and transposon mutagenesis studies by Rawat *et al* had enabled reannotation of MSMEG_4189 as MshC [51]. As a penultimate step in the mycothiol biosynthesis pathway, this enzyme is known to catalyse the activation of cysteine (like a canonical CysRS) which is then transferred to 1D-myo-inosityl-2-amino-2-deoxy-alpha-D-glucopyranoside (GlcN-Ins) in an ATP-dependent reaction unlike the canonical CysRS which transfers activated Cysteine to tRNA^Cys^ [52, 53]. Although, these studies indicated non-essentiality of MshC to the survival of *M*. *smegmatis*, the inability to knockout its homolog in *M*. *tuberculosis* Erdman strain [54] suggested differences in the essentiality of the same enzyme function in two related species. An independent mutagenesis study had suggested canonical CysRS of *M*. *tuberculosis* to be essential [32], thereby, suggesting that MshC and CysRS could not complement each other’s function. We decided to investigate the essentiality of CysRS by employing a conditional expression strain to see if the situation is similar in *M*. *smegmatis*.

### Sequence and transmembrane segment analysis of lysyl-tRNA synthetase

The two Gram negative and the three mycobacterial species analysed in this study seem to code for two LysRS proteins each, while the two Gram positive bacteria code for a single LysRS gene each (Table 2). Among the two LysRS of *E*. *coli*, LysS is constitutively expressed, while LysU expression was found to be inducible by heat and other stress factors [21]. A third putative Lysyl-tRNA synthetase encoded by *epmA* was shown to lysylate the elongation factor P (EF-P) and hence the annotation, elongation factor P Lys34-lysyltransferase [55]. The protein sequence alignment of all three LysRS of *E*. *coli* indicated that *epmA* gene product has about 30% identity with the other two LysRS which among themselves have about 90% identity. However, Yanagisawa *et al*., were able to demonstrate its structural features resembled those of class II aaRS [55]. At the primary structure level, the *lysS* and *genX* products of *H*. *influenzae* were found to be about 70% and 62% identical to that of *lysS* and *epmA* of *E*. *coli* respectively. In addition to Lys-tRNA^Lys^ synthesis activity, in many bacteria LysRS was found to be the most efficient among the various aaRS to synthesize diadenosine tetraphosphate (Ap_4_A) and its analogues. These molecules which accumulate in *E*. *coli* immediately after heat shock or oxidative stress coinciding with increased LysU expression were suggested to play the role of signalling molecules (alarmones) to modulate the stress response [56, 57, 21]. In eukaryotes, ApnA binding proteins include but not limited to haemoglobin, glyceraldehyde-3-phosphate dehydrogenase, DnaK, ClpB, glycogen phosphorylase and P2-purinergic receptors [58].

Genes encoding LysRS from three mycobacterial species, *H*. *influenzae* and *E*. *coli* are listed in Table 3. The lengths of LysRS protein indicated the presence of two types of lysyl-tRNA synthetases in mycobacteria, one with about 500 amino acids (group A) and the other with about 1000 amino acids (group B).

**Table 3:** List of lysyl tRNA synthetase genes*.

Description	Length(amino acids)	Genename	Organism
lysS; lysine-tRNA synthetase,constitutive (EC:6.1.1.6); class II	505	b2890	*E*. *coli*
lysU; lysine-tRNA synthetase,inducible (EC:6.1.1.6); class II	505	b4129	*E*. *coli*
epmA; Elongation Factor P Lys34 lysyltransferase; lysyl-tRNA synthetase, class II	325	b4155	*E*.*coli*
lysS, lysyl-tRNA synthetase, class II	502	HI1211	*H*. *Influenzae*
genX; lysyl-tRNA synthetase, class II	323	HI0836	*H*. *Influenzae*
lysS; lysyl-tRNA synthetase (EC:6.1.1.6); class II	507	MSMEG_6094	*M*. *smegmatis*
lysS; lysyl-tRNA synthetase (EC:6.1.1.6); class II	1089	MSMEG_3796	*M*. *smegmatis*
lysS; lysyl-tRNA synthetase (EC:6.1.1.6); class II	507	ML_0233	*M*. *leprae*
lysS; lysyl-tRNA synthetase (EC:6.1.1.6); class II	1039	ML_1393	*M*. *leprae*
lysS; Lysyl-tRNA synthetase 1 LysS (EC:6.1.1.6); class II	505	Rv3598c	*M*. *tuberculosis*
lysX; bifunctional lysine-tRNA ligase/phosphatidyl glycerol lysyltransferase (EC:6.1.1.6); lysyl-tRNA synthetase, class II	1172	Rv1640c	*M*. *tuberculosis*

*KEGG genes database

A high degree of similarity with about 80% identity was observed within the group A and within the group B mycobacterial LysRS when their amino acid sequences were aligned (Table 3). However, alignment of group A LysRS (shorter) sequence with that of group B LysRS (longer) revealed that the C-terminal half of group B sequence (from 600^th^ amino acid till the end) had significant homology (about 47% identity) along the entire length of group A protein. Fig E in S1 File represents one such analysis using the *M*. *smegmatis* LysRS (MSMEG_3796 and MSMEG_6094) sequences. About 40% sequence identity was observed for group A LysRS and for C-terminal half of group B LysRS with *E*. *coli* LysS suggesting that in mycobacteria, group A enzymes (MSMEG_6094, ML_0233, Rv3598c) are the cognate LysRS and the C-terminal domain of group B enzymes (MSMEG_3796, ML_1393, Rv1640c) also harbour the lysyl-tRNA synthetase activity.

The LysX of *M*. *tuberculosis*, belonging to group B LysRS has been described to possess an additional function. Maloney *et al*. have shown that the N-terminal half of this protein is involved in the transfer of lysine moiety to phosphatidyl glycerol (PG) to synthesize lysinylated phosphatidyl glycerol (L-PG) which confers resistance to cationic anti-microbial peptides (CAMPs). Maloney *et al*. had also shown that the intact LysX protein with both domains are required for the synthesis of L-PG, i.e., the N-terminus of *M*. *tuberculosis* LysX codes for MprF (multiple peptide resistance factor) like protein which catalyses the transfer of lysyl moiety to PG from Lys-tRNA^Lys^ synthesized through the action of C-terminal half of LysX. The gene disruption studies had demonstrated that although LysX is not necessary for *in vitro* growth of *M*. *tuberculosis*, it is needed during the course of infection to efficiently counter the effect of host produced CAMPs [59]. By virtue of being very similar in its primary structure and possibly secondary and tertiary structures to *M*. *tuberculosis* LysX, MSMEG_3796 probably performs a similar function in *M*. *smegmatis*. A primary structure alignment of N-terminal domain of MSMEG_3796 with MprF (encoded by *fmtC*) of *S*. *aureus* revealed an overall 23% identity similar to that seen with N-terminal domain of *M*. *tuberculosis* LysX. Thus, the additional LysRS present in mycobacteria was found to be different from the additional CysRS in that it activates the cognate amino acid, transfers the activated amino acid moiety to cognate tRNA^Lys^, and subsequently uses this charged tRNA as a substrate to transfer the lysyl group to PG.

**Fig 2:**
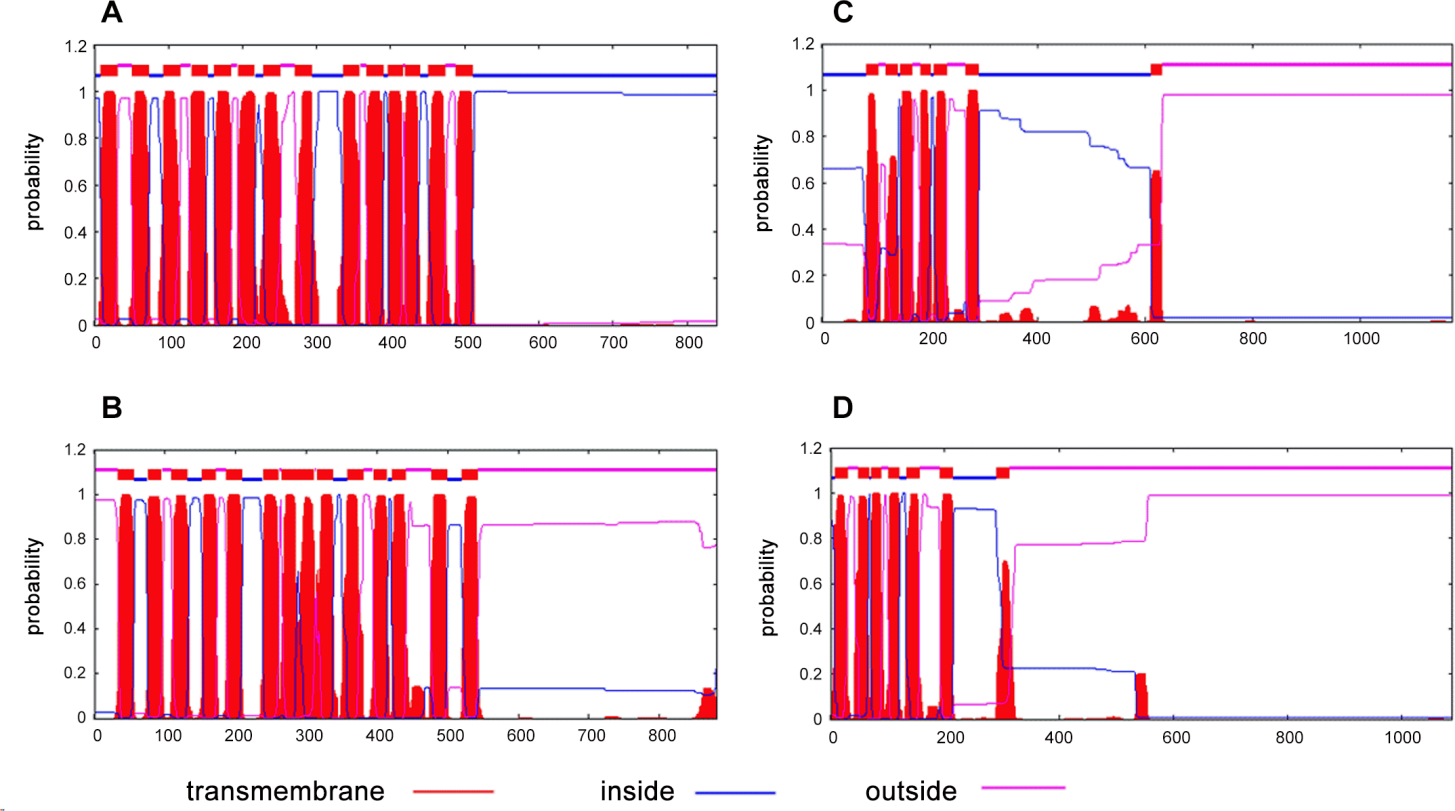
Transmembrane segment prediction analysis. TMHMM plots for *S*. *aureus* MprF (**A**), *P*. *aeruginosa* PA0290 (**B**), *M*. *tuberculosis* LysX (**C**) and MSMEG_3796 (**D**).

*S*. *aureus* MprF, a virulence factor was shown to confer resistance to cationic antimicrobial peptides (CAMPs) like defensins [60]. Homologs of *fmtC* gene have been found in other pathogenic organisms like *P*. *aeruginosa* and *E*. *faecalis* as well [61]. Unlike the mycobacterial LysX, the MprF protein lacks a lysyl-tRNA synthetase domain and is believed to use the Lys-tRNA^Lys^ from the cellular pool to transfer the lysine moiety onto PG. The membrane bound MprF protein was shown to contain two domains with the lysyl transferase activity at its C-terminal half and a flippase domain which is required to flip the newly synthesized L-PG across the membrane at its N-terminal half. Ernst *et al*., had demonstrated that these two domains can be expressed independently of each other and still bring about the synthesis and translocation of L-PG across the membrane [62]. This study also showed that N-terminal domain of MprF contains up to 14 transmembrane segments of which the first eight segments are sufficient to catalyse the flipping of L-PG. A re-examination of the pairwise alignment of MSMEG_3796 with *S*. *aureus* MprF indicated that the homologous region between them lies from 234^th^ to 530^th^ amino acid of MSMEG_3796. Alignment of MSMEG_3796 with MSMEG_6094 had indicated that the segment from 600^th^ to 1080^th^ amino acid of MSMEG_3796 possesses lysyl-tRNA synthetase activity (Fig E in S1 File). This leaves its N-terminal 230 amino acids segment with no assigned function. BlastP analysis of the N-terminal 230 amino acids of MSMEG_3796 did not yield any sequences with significant homology. We performed a transmembrane segment (TMS) prediction analysis of MSMEG_3796 and *M*. *tuberculosis* LysX sequences using TMHMM tool to see if their extreme N-terminal regions had any transmembrane segments similar to the ones in *S*. *aureus* MprF. Fig 2 shows the transmembrane segments as predicted by TMHMM for all the proteins analysed. This data indicated the presence of 5–6 transmembrane segments in both *M*. *tuberculosis* LysX (Fig 2C) and MSMEG_3796 (Fig 2D) at their extreme N-terminal region spanning about 200 amino acids. Although, this may be sufficient to keep them membrane bound, it remains to be experimentally proven whether these 5–6 TMs are sufficient for flipping the newly synthesized L-PG across the mycobacterial membrane as demonstrated by Ernst *et al*., for *S*. *aureus* MprF [62]. Interestingly, both the mycobacterial proteins were found to possess membrane spanning segments despite the absence of any homology at the primary structure level with the similar region from *S*. *aureus* MprF.

Similar to the additional activities found with bacterial LysRS, the human homolog (KRS) was also shown to have additional function where it triggers the dissemination of cancer cells from the primary tumour when it associates itself with the plasma membrane. Nam *et al*., had demonstrated that KRS is indeed involved in the intracellular signal transduction resulting in invasive dissemination of colon cancer spheroids [63] and suggested that it could serve as a suitable target for the development of anti-metastatic therapy.

Maloney *et al*., had confirmed that LysX is not essential for the *in vitro* survival of *M*. *tuberculosis* and that LysS could not complement the absence of lysyl-tRNA synthetase function from LysX [59]. Conversely, the transposon mutagenesis study in *M*. *tuberculosis* had indicated LysS to be *in vitro* essential and that LysX could not complement its function [32]. The essentiality data could be very similar in *M*. *smegmatis*, however, the absence of direct experimental evidence triggered us to generate and evaluate conditional expression strains of both MSMEG_3796 and MSMEG_6094 to assess their essentiality.

### LeuRS is essential for the growth of *M*. *smegmatis*

Conditional expression strains which use regulated expression system offer the flexibility of assessing gene essentiality under a variety of growth conditions. The inability of conditional expression strain to grow in the absence of added inducer would indicate the essentiality of a gene under investigation. However, a tightly regulated inducible expression system is a basic necessity to derive unambiguous conclusion regarding the essentiality of a target gene under investigation. Acetamide and tetracycline regulated systems have been employed earlier for this purpose in mycobacteria [64–67]. We and others have reported the successful application of a pristinamycin-inducible system for conditional expression of genes in both *M*. *smegmatis* and *M*. *tuberculosis* [26, 27, 68]. Recently, we had reported the generation and validation of an IPTG-inducible conditional expression system for mycobacteria [25]. Using this system, we confirmed the essentiality of several clinically validated targets in mycobacteria. The assessment of essentiality of *M*. *smegmatis* leucyl-tRNA synthetase was performed as a validation step prior to evaluating the essentiality of CysRS and LysRS. There were multiple reasons for the selection of LeuRS as a validation tool: (1) it is a single gene in *M*. *smegmatis* with no known redundancy, (2) it belongs to the same family of proteins as others being investigated in this study, (3) it is a single gene in its transcription unit and hence its disruption is unlikely to cause any downstream polar effects, (4) it is highly homologous (about 80% identity) to its counterpart in *M*. *tuberculosis* which has been shown to be essential via genetic and chemical means [32, 12].

**Fig 3:**
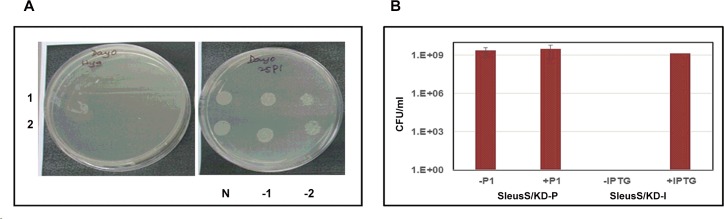
Inducer dependency for the growth of *M*. *tuberculosis* and *M*. *smegmatis* LeuRS conditional expression strains. Several confirmed recombinant colonies were grown in the presence of inducer till they reached mid-log phase, cells were washed, resuspended in fresh 7H9 broth and the cultures were either spotted or various dilutions plated for enumerating colony forming units (CFUs). Plates were incubated at 37°C for 28 days for *M*. *tuberculosis* strains and 48 hours for *M*. *smegmatis* strains, respectively. (**A**). Recombinant colonies (1 and 2) of TleuS/KD-P analysed for growth in the absence (left) and the presence (right) of P_**1**_; N, -1 and -2 are the undiluted, 10^−1^ and 10^−2^ dilutions. (**B**). Culture dilutions of SleuS/KD-P and SleuS/KD-I strains were plated with and without P_**1**_ and IPTG, respectively. Bars in the graph represent CFU/ml calculated from the colony numbers that appeared on plates under each of the growth condition specified.

In order to compare the data across the two species, we generated conditional expression strains of LeuRS of both *M*. *tuberculosis* and *M*. *smegmatis* using the pristinamycin-inducible expression system. The recombinant strains, SleuS/KD-P and TleuS/KD-P were grown in the presence of 300 ng/ml of pristinamycin 1 (P_1_) till they reached mid-log phase. The cells were washed 3 times with plain 7H9 broth and spotted on 7H11 plates without and with different concentrations of P_1_ to assess their inducer dependency for growth. All the recombinant TleuS/KD-P colonies tested, showed an absolute P_1_ dependency for growth confirming its essentiality for the growth of *M*. *tuberculosis in vitro*. Fig 3A represents the results for two independent colonies which showed no growth in the absence of P_1_ but showed a good colony morphology at 25 ng/ml P_1_. However, the *M*. *smegmatis* recombinant colonies grew equally well whether or not P_1_ was present in the growth medium (data not shown). To confirm this result, one of the SleuS/KD-P recombinant colonies was grown and processed as described earlier to prepare an inoculum for plating several dilutions on 7H11 plates with and without P_1_. We hypothesized that the growth of a well isolated colony from a diluted culture would demonstrate cleaner inducer dependency than the culture spots from a broth culture. However, the strain showed no difference in the growth phenotype whether or not P_1_ was present in the plates (Fig 3B) suggesting either the pristinamycin system is leaky in *M*. *smegmatis* or LeuRS is not essential for *M*. *smegmatis*. On the other hand, SleuS/KD-I strain could not grow unless IPTG was provided in the growth medium, suggesting the essentiality of LeuRS (Fig 3B). The data from the two inducible systems were contradicting, however, we decided to consider the data from the IPTG-inducible system to be more reliable because of the tightness in regulation of expression it had demonstrated in the previous study [25] and for the reasons stated earlier. The results also suggested that the pristinamycin-inducible system is probably not as well-regulated in *M*. *smegmatis* as in *M*. *tuberculosis*. Therefore, we decided to employ the IPTG-inducible system to generate and evaluate conditional expression strains of MSMEG_6074, MSMEG_3796 and MSMEG_6094 to investigate their essentiality.

### Canonical LysRS and CysRS are essential for growth of *M*. *smegmatis*

**Fig 4:**
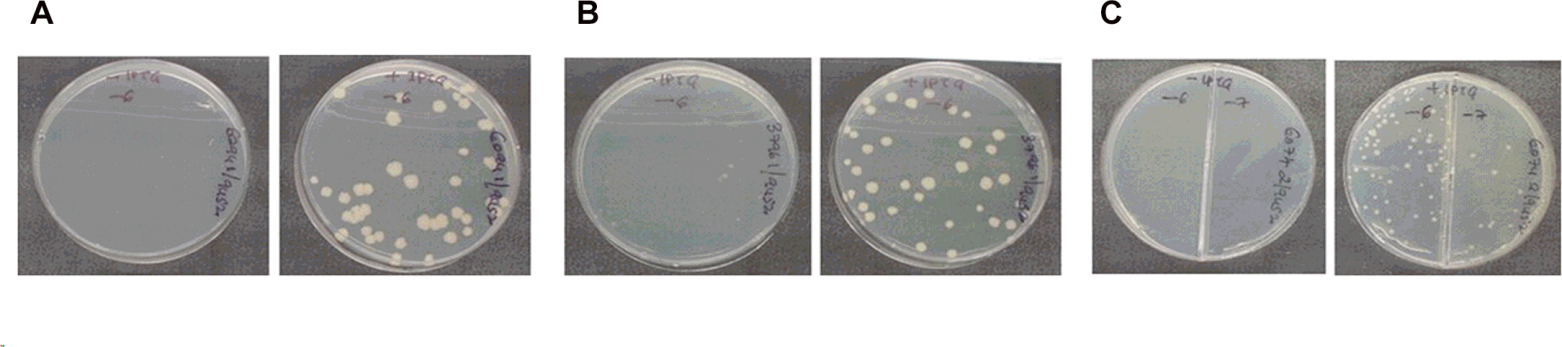
Analysis of inducer dependence for growth. Identified recombinant colonies of S6094/KD-I, S3796/KD-I and ScysS/KD-I were grown with 100 μM IPTG until they reached mid-log phase, washed and the culture suspension in plain 7H9 broth was diluted and plated on plates with and without IPTG. The plates were incubated at 37°C for 48 hours and photographed. In all the cases, plate on left is without IPTG and plate on right is with IPTG. (**A**) S6094/KD-I; (**B**) S3796/KD-I and (**C**) ScysS/KD-I.

Conditional expression strains of *M*. *smegmatis* CysRS (ScysS/KD-I) and both LysRS (S3796/KD-I and S6094/KD-I) were generated using an IPTG-inducible system as described in materials and methods. A minimum inducer concentration requirement test performed by plating the culture at different concentrations of the inducer (0, 5, 10, 50, 100 and 500 μM IPTG) indicated 100 μM IPTG as the optimal concentration for the growth of these strains (data not shown). The strains were then grown in 7H9 broth supplemented with 50 μg/ml hygromycin and 100 μM IPTG till they reached mid-log phase. Inducer free cell suspensions were prepared by washing the cells with fresh broth and used as inoculum to plate on 7H11 plates supplemented with 100 μM IPTG or no IPTG. The plates were observed for growth after 48 hours of incubation at 37°C. Although, there were no colonies on plates without IPTG in the case of S6094/KD-I and ScysS/KD-I, tiny colonies could be seen in the case of S3796/KD-I (Fig 4). All the plates were incubated for an additional 48 hours to see if differences in the phenotype would be more pronounced following an extended period of incubation. While no colonies grew in S6094/KD-I and ScysS/KD-I plates without IPTG after 96 hours, in the case of S3796/KD-I, the tiny colonies observed on plates without IPTG at 48 hours grew into well-formed colonies (data not shown).

**Fig 5:**
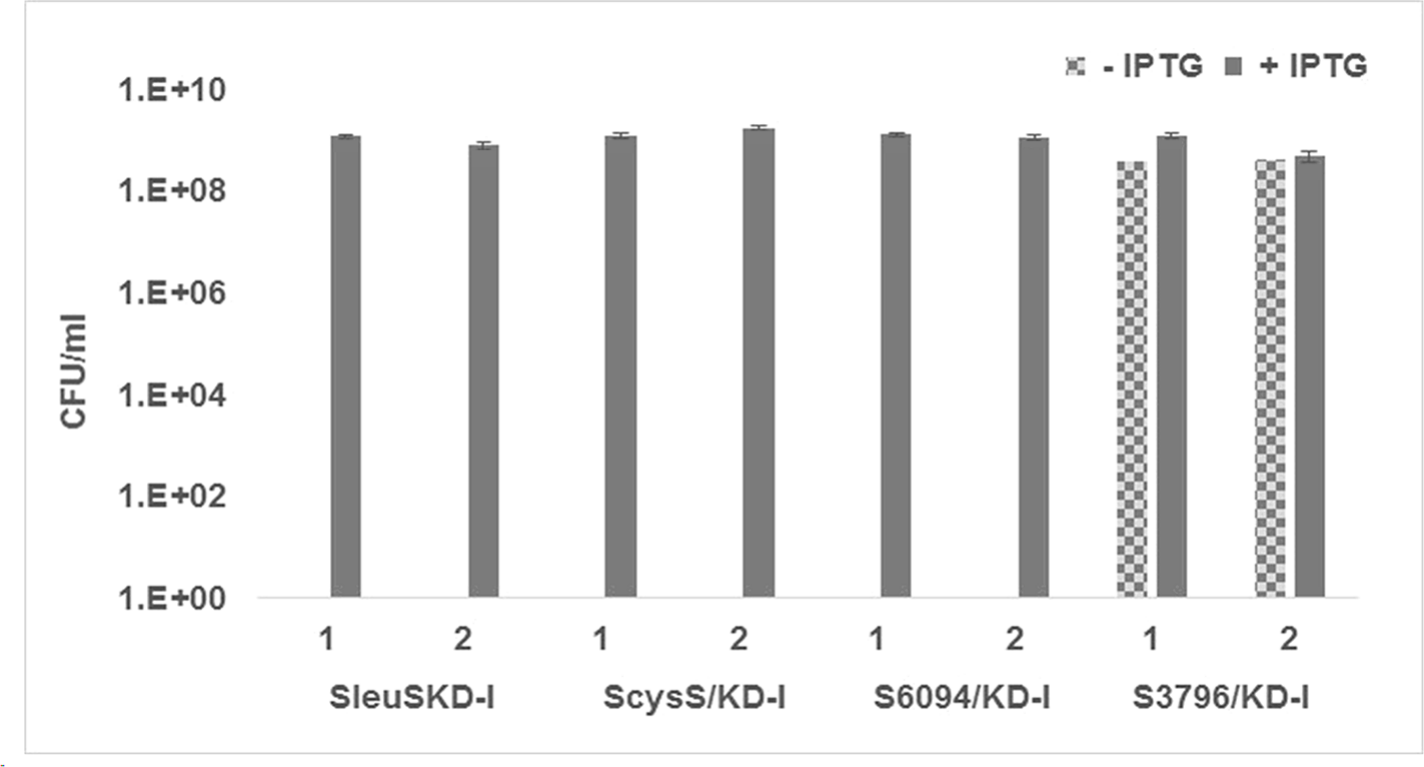
Inducer dependency for growth of *M*. *smegmatis* LeuRS, LysRS and CysRS conditional expression strains. The conditional expression strains SleuS/KD-I, SCysS/KD-I, S6094/KD-I and S3796/KD-I were grown in the presence of 100 μM IPTG till they reached mid-log phase, the cells were harvested, washed to remove traces of inducer and resuspended in fresh 7H9 broth to be used as inoculum. Several dilutions of these cultures were plated on 7H11 plates with and without IPTG. Plates were incubated at 37°C for 96 hours and the colonies were counted both at the end of 48 hours and 96 hours of incubation.

The delayed growth phenotype of S3796/KD-I conditional expression strain in the absence of inducer was different from that observed by Maloney *et al*., [59] with *M*. *tuberculosis* LysX knockout strain which didn’t show any growth defect *in vitro*. In order to confirm the results, the experiment was repeated with two colonies for each of the conditional expression strain. Two colonies of *M*. *smegmatis* LeuRS conditional expression strain were also included in the experiment. Dilutions of the culture suspensions prepared as described in materials and methods were plated on 7H11 plates supplemented with 100 μM IPTG or no IPTG. At the end of 48 hours of incubation, conditional expression strains showed the same phenotype as described earlier. After 96 hours of growth, the colonies appearing on each plate were counted, the CFU/ml calculated and plotted against the growth condition for each of the strain. While the SleuS/KD-I, S6094/KD-I, and ScysS/KD-I strains grew only in the presence of IPTG, S3796/KD-I strain showed growth even in the absence of IPTG at 96 hours indicating the reproducibility of the earlier observation (Fig 5). These results further confirm the essentiality of *M*. *smegmatis* LeuRS, CysRS, MSMEG_6094 and the non-essentiality of MSMEG_3796.

## Summary and Conclusion

A search for genes encoding aminoacyl-tRNA synthetases of *M*. *smegmatis* in KEGG genes database yielded 24 entries. Analysis of the primary structures of some of the additional proteins—TilS, Glu-Q-RS, Prolyl-tRNA editing protein (YbaK homolog), MshC and MSMEG_3796 (LysX homolog) of *M*. *smegmatis* with their orthologs from *E*. *coli* and / or *M*. *tuberculosis* followed by literature survey enabled understanding their physiological roles. Although non-essential, it remains unclear and confusing why Glu-Q-RS is conserved across different bacterial genera when its activity could lead to the synthesis of several proteins with conserved changes–glutamate for aspartate. The essential nature of the activities catalysed by TilS and prolyl-tRNA editing protein substantiated their retention. The conservation of MshC and LysX homolog across different mycobacterial species indicated the importance of their function for mycobacterial survival.

The dispensability of MSMEG_3796 and essentiality of the canonical CysRS and LysRS for the survival of *M*. *smegmatis* could be established using their conditional expression strains. The *in vivo* essentiality in the pathogenic mycobacteria and the *in vitro* non-essentiality of LysX homologs in the pathogenic and saprophytic mycobacteria could be readily explained based on the observation that LysX is required to counter the cationic anti-microbial peptides the bacteria would encounter within their host. However, reasons for the differential essentiality of MshC in the pathogenic versus the saprophytic mycobacteria is not clear as it has been demonstrated to confer mycobacteria the ability to resist several alkylating agents and anti-mycobacterials [51].

The intriguing fact about MSMEG_3796 and MSMEG_6094 was that despite having the entire LysRS domain in both these proteins, they could not complement each other’s absence. The Lys-tRNA^Lys^ synthesized by each of these enzymes seems to get utilized towards different physiological functions, L-PG synthesis and protein synthesis, respectively. The difference in the localization of these proteins could be the reason for this non-redundant function. Bioinformatics analysis enabled us to predict the presence of transmembrane segments in MSMEG_3796 suggesting that it could be membrane bound while MSMEG_6094, a homolog of canonical LysRS required for translation process could be cytosolic. It is highly likely that the membrane bound MSEMG_3796 cannot access the Lys-tRNA^Lys^ synthesized by cytosolic MSMEG_6094 and vice versa. On the other hand it would be interesting to identify the factor which has enabled the membrane bound MprF in *S*. *aureus* to utilize the LystRNA^Lys^ synthesized by the cytosolic LysRS

This study also enabled us to notice several interesting facts about aminoacyl-tRNA synthetases and their homologs. A number of them were found to be involved in non-translational functions such as synthesis of diadenosine tetraphosphate (Ap_4_A) and analogues in several bacteria, synthesis of MshC and LP-G in mycobacteria, lysinylation of elongation factor by epmA/genX products in Gram negatives and involvement in cardiovascular development, immune response, signalling events as in triggering metastatic events in human cancer [69, 70].

Thus, the literature and bioinformatics analysis along with 3-D structure modelling enabled us to understand the likely functions of the ‘additional’ aminoacyl-tRNA synthetases found in *M*. *smegmatis*. The information gathered from this study also indicated the unique activity of these ‘additional’ aminoacyl-tRNA synthetases and very importantly the absence of redundancy in the function of canonical aaRS despite the presence of common functional domains. These studies have thus offered valuable insights into the role of various aminoacyl-tRNA synthetases in the growth and survival of mycobacteria which in turn could provide avenues for further research into the design of specific anti-mycobacterials agents.

## Supporting Information

S1 File Plasmid maps of pAZI9452 and pAZI947 (Fig A). List of primers used in the study (Table A). Genomic organization in the conditional expression strain (Fig B). Generic reaction scheme for aminoacyl-tRNA synthetases (Fig C). The VERIFY-3D Average score of modelled MSMEG_5671 structure (Fig D). Homology among *M*. *smegmatis* lysyl-tRNA synthetases (Fig E). (DOCX)
