# Supplementary material for: Essentiality Assessment of Cysteinyl and Lysyl-tRNA Synthetases of Mycobacterium smegmatis
Source: PLoS One. 2016 Jan 21;11(1):e0147188. doi: 10.1371/journal.pone.0147188 (PMC4721953; doi:10.1371/journal.pone.0147188)
Supplement: S1 File — (DOCX) [file pone.0147188.s001.docx]

**Essentiality assessment of**

**Cysteinyl and Lysyl-tRNA synthetases of**

***Mycobacterium smegmatis***

Sudha Ravishankar,^1*#a^ Anisha Ambady,^1#b^ Rayapadi G Swetha,^2#a^ Anand Anbarasu,^2#a^ Sudha Ramaiah,^2#a^ Vasan K. Sambandamurthy,^1#c^

^1^AstraZeneca India Pvt Ltd, Bellary Road, Hebbal, Bengaluru, India 560024

^2^School of Biosciences & Technology, VIT University, Vellore, India 632014

*Corresponding author

[mailsuravi01@gmail.com](mailto:mailsuravi01@gmail.com)

**Supporting Information S1 File:**

**Figure A.**


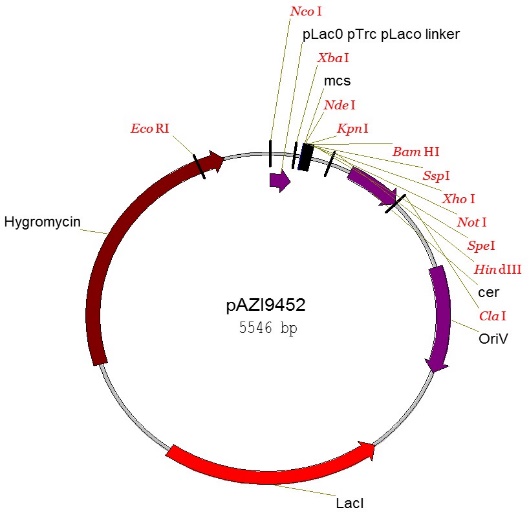

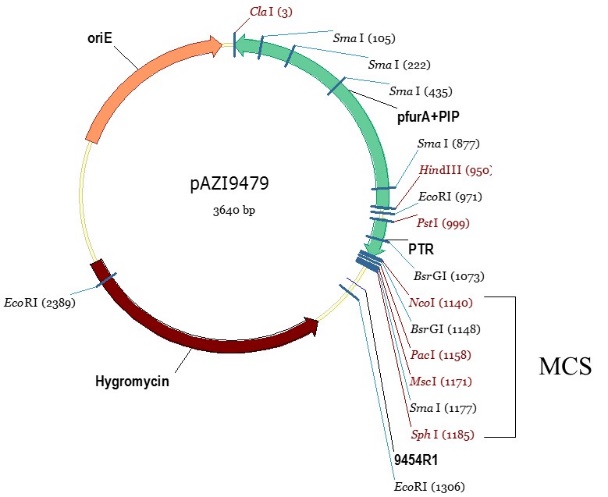


**Plasmid maps of pAZI9452 and pAZI9479.** Conditional expression plasmids with IPTG inducible promoter system (pAZI9452) and pristinamycin inducible promoter system (pAZI9479).

**Table A: List of primers used in the study**

| **Primer name** | | **Sequence (5’----3’)** | |
| --- | --- | --- | --- |
| SleuS9452F | | TTATCTCATATGGTGACCGAACCCGCAACCACG | |
| SleuS9479F | | TTATCTCCATGGGAACCGAACCCGCAACCACG | |
| SleuS9452R | | CTAGAAGCTTCCGGAACACCGGGAAGTTGC | |
| SLeuSScrnF | | GTCTTGGGAGGTCAGCGACTTC | |
| SLeuSScrnR_ | | GGTGATGCGCATCATCCACTGG | |
| S6074_9452F | | ATCTTTCATATGACCGATCGCGCTCAAGC | |
| S6074_9452R | | TAGTAAGCTTAATCCATGCCGCCTGCGTGG | |
| S6074ScrnF | | GGTCGGTGATGCAAAGTAGC | |
| S6074ScrnR | | CTCGTTCTCGTGGTGCGG | |
| S6073complF | | ATCTGGATCCAATGGCCGGAAATTCACAGCGGC | |
| S6073complR | | AGATAAGCTTCAATGATGATGATGATGATGGCTGCGGCGCTGACG | |
| S6094_9452F | | ATCTCTCATATGACCCCAGCCGATCGTGACG | |
| S6094_9452R | | CTGACGAAGCTTAGATCCTCAGGTACAGATC | |
| S6094ScrnF | | GTTCCGAGGTGCGTTCACGC | |
| S6094ScrnR | | GCGAAAGTTGCGATTCAACTCGAA | |
| S3796_9452F | | ATCTATCATATGGTGGCCTGGGTGCCCG | |
| S3796_9452R | | TTGATGAAGCTTAGCCGTAGAGCTCGAGCAG | |
| S3796ScrnF | | CTATGACCGTAATCAGTCGC | |
| S3796ScrnR | | GGCACACGCCGACCTCGAC | |
| 9452R1 | | GAACCTGCAATTAGCCCTTAGGA | |
| 9452F1 | | TTTTGTTTAACTTTAAGGAGGAGATAT | |
| 0318R1 | | CCAGGAGTCGTCGCCACCAAT | |
| 0318F2 | | TTGATGGTACCGAGCTCGAATTC | |
| TleuS9452F | | ATATCCATGGGAACCGAATCGCCAAC | |
| TleuS9452R | ATATTGGCCATCACGGAAAATTGCCCC | |  |

**Figure B.**

**
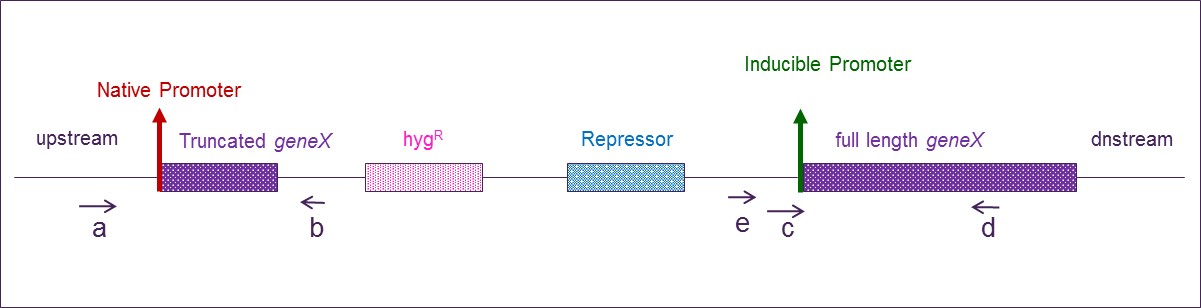
**

**Genomic organization in the conditional expression strain.** Strain is generated through single cross over recombination between the wild-type genome and a suicide conditional expression plasmid with about 700 bp of *geneX* cloned downstream of inducible promoter. a, b, c, d and e are primers for PCR screening of conditional expression strain. Arrows above these primers indicate the primer 5’ 3’ direction.

**Figure C.**

**Generic reaction scheme for aminoacyl-tRNA synthetases.**

**Figure D**

**
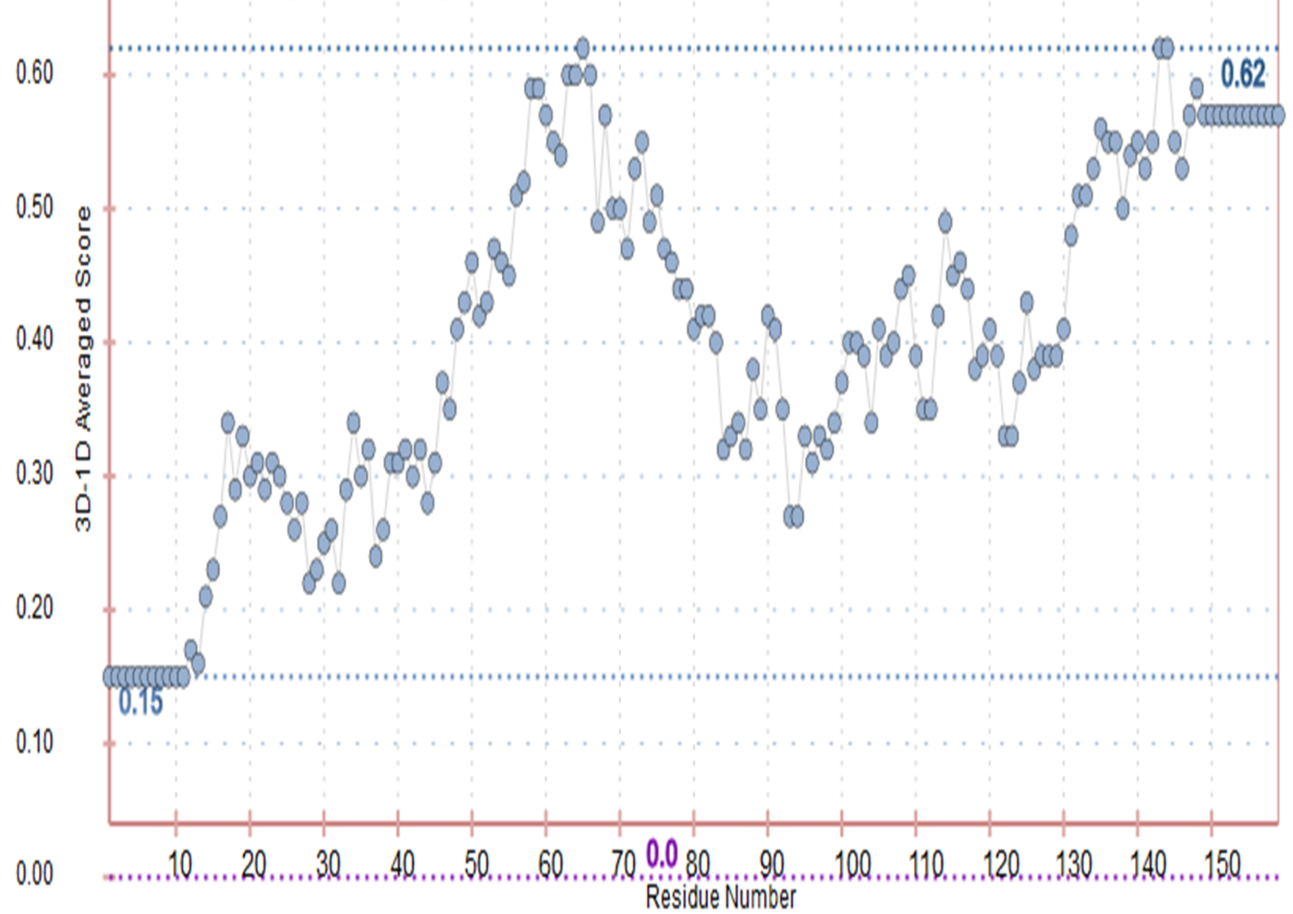
**

**The VERIFY-3D Average score of modelled MSMEG_5671 structure.** The best 3-D structure model of MSMEG_5671 produced by I-TASSER was run through VERIFY-3D program for quality analysis.

**Figure E**

**
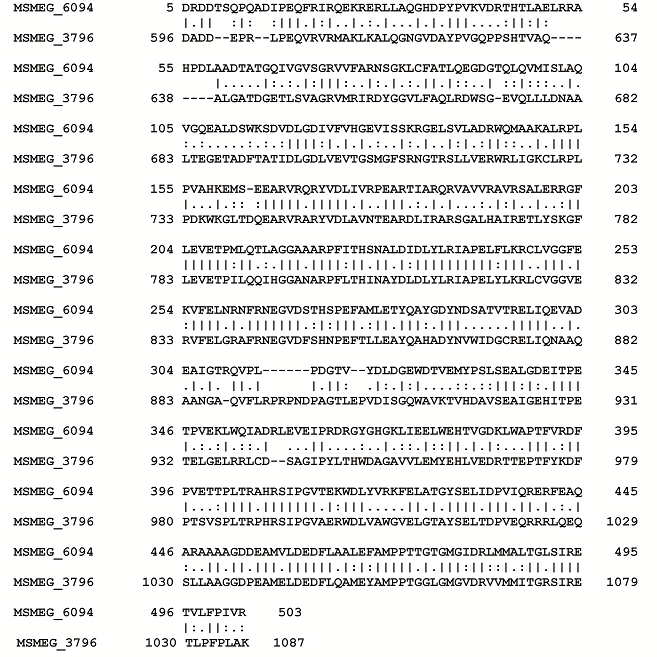
**

**Homology among *M. smegmatis* lysyl-tRNA synthetases.** Alignment of the two LysRS (MSMEG_3796 and MSMEG_6094) from *M. smegmatis*.
